# The assessment of risk factors for long-term survival outcome in ypN0 patients with rectal cancer after neoadjuvant therapy and radical anterior resection

**DOI:** 10.1186/s12957-021-02262-x

**Published:** 2021-05-21

**Authors:** Marcin Zeman, Marek Czarnecki, Ewa Chmielik, Adam Idasiak, Władysław Skałba, Mirosław Strączyński, Piotr J. Paul, Agnieszka Czarniecka

**Affiliations:** 1 The Oncologic and Reconstructive Surgery Clinic, Maria Sklodowska-Curie National Research Institute of Oncology, Gliwice Branch, Wybrzeze Armii Krajowej 15, 44-100 Gliwice, Poland; 2 Tumor Pathology Department, Maria Sklodowska-Curie National Research Institute of Oncology, Gliwice Branch, Wybrzeze Armii Krajowej 15, 44-100 Gliwice, Poland; 3 II Clinic of Radiotherapy and Chemotherapy, Maria Sklodowska-Curie National Research Institute of Oncology, Gliwice Branch, Wybrzeze Armii Krajowej 15, 44-100 Gliwice, Poland; 4 Department of Pathology, Institute of Medical Sciences, University of Opole, Oleska 48, 45-052 Opole, Poland

**Keywords:** Stage migration, Rectal neoplasms, Lymph node yield, Charlson comorbidity index, Late anastomotic leak, Anterior rectal resection

## Abstract

**Background:**

The main negative prognostic factors in patients with rectal cancer after radical treatment include regional lymph node involvement, lymphovascular invasion, and perineural invasion. However, some patients still develop cancer recurrence despite the absence of the above risk factors.

The aim of the study was to assess clinicopathological factors influencing long-term oncologic outcomes in ypN0M0 rectal cancer patients after neoadjuvant therapy and radical anterior resection.

**Methods:**

A retrospective survival analysis was performed on a group of 195 patients. We assessed clinicopathological factors which included tumor regression grade, number of lymph nodes in the specimen, Charlson comorbidity index (CCI), and colorectal anastomotic leakage (AL).

**Results:**

In the univariate analysis, AL and CCI > 3 had a significant negative impact on disease-free survival (DFS), disease-specific survival (DSS), and overall survival (OS). After the division of ALs into early and late ALs, it was found that only patients with late ALs had a significantly worse survival. The multivariate Cox regression analysis showed that CCI > 3 was a significant adverse risk factor for DFS (HR 5.78, 95% CI 2.15–15.51, *p* < 0.001), DSS (HR 7.25, 95% CI 2.25–23.39, *p* < 0.001), and OS (HR 3.9, 95% CI 1.72–8.85, *p* = 0.001). Similarly, late ALs had a significant negative impact on the risk of DFS (HR 5.05, 95% CI 1.97–12.93, *p* < 0.001), DSS (HR 10.84, 95% CI 3.44–34.18, *p* < 0.001), and OS (HR 4.3, 95% CI 1.94–9.53, *p* < 0.001).

**Conclusions:**

Late AL and CCI > 3 are the factors that may have an impact on long-term oncologic outcomes. The impact of lymph node yield on understaging was not demonstrated.

**Supplementary Information:**

The online version contains supplementary material available at 10.1186/s12957-021-02262-x.

## Background

Long-term treatment results of rectal cancer patients after the introduction of combined treatment regimens and the techniques of total mesorectal excision have significantly improved [1]. After neoadjuvant therapy and radical surgery, negative prognostic factors include regional lymph node involvement and a number of histopathological factors related to the potential for tumor invasiveness, such as lymphovascular invasion (LVI), perineural invasion (PNI), poor differentiation, or the mucinous component of the tumor [2, 3]. However, even some ypN0M0 patients with no additional histopathological risk factors have a relapse. Several studies showed a negative impact of colorectal anastomotic leakage (AL) on long-term survival after anterior rectal resection (AR) [4, 5]. However, such findings were not confirmed in all studies [6]. Additionally, the minimum number of lymph node yield (LNY) that could allow to avoid understaging in ypN0 patients has not been established either. Research found either a negative impact [7, 8] or no impact [9, 10] of low LNY on long-term oncologic outcomes. The influence of preoperative radiotherapy on the reduction in the number of resected lymph nodes was also demonstrated [11]. Some studies found that low LNY was associated with a good response to neoadjuvant therapy [12]. Few studies suggested that apart from the effect associated with increased mortality due to comorbidities, their direct impact on the course of the neoplastic disease was possible. However, the mechanism of this interaction has not been fully understood yet [13].

The aim of the study was to assess the selected clinicopathological factors influencing long-term oncologic outcomes in ypN0M0 rectal cancer patients after neoadjuvant therapy and radical AR in the group of patients with a good prognosis without major histological risk factors.

## Methods

### Patients

Between 2008 and 2016, 328 radical (R0) ARs were performed at the National Research Institute of Oncology in Gliwice, Poland, in rectal cancer patients after neoadjuvant therapy without synchronous distant metastases. Metastases that occurred within 3 months after surgery were considered synchronous. Prior to treatment, all patients had been staged T3N0 or T1-3N+. The process of selecting the low-risk study group is given in the flowchart (see Additional file 1). Finally, 195 patients (82 females, 113 males) without main risk factors were enrolled in a retrospective study. Patient characteristics are given in Table 1.

**Table 1:** Patient characteristics

		n
Sex	Females	82 (42.1%)
Age	Mean (SD)	64 (10.03)
BMI (kg/m2)	Mean (SD)	26.21 (4.03)
CCI	2	142 (72.8%)
	3	42 (21.6%)
	> 3	11 (5.6%)
Clinical stage prior to treatment	II	58 (29.7%)
	III	137 (70.3%)
Neoadjuvant therapy	RT	127 (65.1%)
	CRT	68 (34.9%)
Time RT-S > = 6 weeks	Yes	107 (54.9%)
Distance from the anal verge (cm)	1–5	57 (29.2%)
	6–10	93 (47.7%)
	11–15	45 (23.1%)
Loop ileostomy	Yes	41 (21%)
yG	1	16 (8.2%)
	2	111 (57.2%)
	x	67 (34.5%)
ypT	0	17 (8.7%)
	1-2	98 (50.3%)
	3	80 (41%)
LNY	Mean (SD)	12.16 (6.01)
LNY groups	1–7	45 (23.1%)
	8–12	72 (36.9%)
	> 12	78 (40%)
Width of the distal margin (cm)	Mean (SD)	2.13 (1.41)
TRG	0–1	91 (46.7%)
	2–3	104 (53.3%)
Anastomotic leakage	No	158 (81%)
	Early	22 (11.3%)
	Late	15 (7.7%)
Adjuvant chemotherapy	Yes	16 (8.2%)

*SD* standard deviation, *BMI* body mass index, *CCI* Charlson comorbidity index, *G* tumor grade, *RT* radiotherapy, *CRT* chemoradiotherapy, *Time RT-S* time from completion of radiotherapy to surgery, *LNY* lymph node yield, *TRG* tumor regression grade

### Procedures

All patients were given neoadjuvant therapy, i.e., radiotherapy (RT) or chemoradiotherapy (CRT). In the RT group, the total dose was 25–42 Gy, while in the CRT group it was 42–54 Gy combined with 1 or 2 cycles of 5-fluorouracil-based chemotherapy. Tumor regression grade (TRG) was based on the assessment of the degree of fibrosis compared to the residual tumor tissue and ranged from 0 to 3, i.e., 0 (complete response), 1 (< 10% residual tumor), 2 (10–50%), and 3 (> 50%). The procedure was performed by laparotomy using the total mesorectal excision technique. End-to-end intestinal anastomosis was performed with a circular stapler. According to the International Study Group of Rectal Cancer (ISREC), AL was defined as a defect of the intestinal wall at the anastomotic site, which resulted in a communication between the intra- and extraluminal compartments and/or the presence of a pelvic abscess near the anastomotic site [14]. All ALs were confirmed radiologically. A CT scan with rectal contrast was performed when symptoms suggestive of possible AL were present [15]. AL diagnosed within 30 days postoperatively was considered early, whereas AL diagnosed after 30 days postoperatively was regarded as late. The severity of comorbidities was assessed based on the original Charlson comorbidity index (CCI) [16] (see Additional file 2).

### Variables

The factors which were analyzed in terms of their impact on survival included sex, age, body mass index (BMI), body surface area (BSA), CCI, clinical stage prior to treatment, type of neoadjuvant therapy (RT vs CRT), time from RT to surgery, rectal tumor location, loop ileostomy (LI), tumor grade (G), ypT, LNY, TRG, width of the distal margin, length of the resected intestine, occurrence of AL with the division into early and late ALs, and post-surgical adjuvant chemotherapy.

### Statistical methods

The survival analysis was performed using the Kaplan-Meier method with the log-rank test. The multivariate analysis was performed using the Cox regression (proportional hazard model). The chi^2^ test was used to assess differences in event rates between the groups. All calculations were made using the statistical package R version 3.6.0.

## Results

AL was postoperatively found in 37/195 (19%) cases, including 22/37 (59.5%) early and 15/37 (40.5%) late ALs. Four patients with early ALs (4/22; 18.2%) and 2 patients with late ALs (2/15; 13.3%) underwent loop ileostomy (LI) at the time of primary surgery (chi^2^ test, *p* = 0.7). The time from surgery to the diagnosis of early and late ALs was 3–27 days (mean 7.7 days) and 36–650 days (mean 137 days), respectively. The severity of ALs according to the ISREC is given in Table 2. The mean LNY was 12.15 (range 1–37, SD 6.01) and the median was 11 (IQR 8–16). The mean follow-up of the study group was 69 months.

**Table 2:** Grading of ALs according to the International Study Group of Rectal Cancer

	Grade A	Grade C
Early ALs (*n* = 22)	2 (9.1%)	20 (90.9%)
Late ALs (*n* = 15)	4 (26.7%)	11 (73.3%)

*AL* Anastomotic leakage

**Fig. 1:**
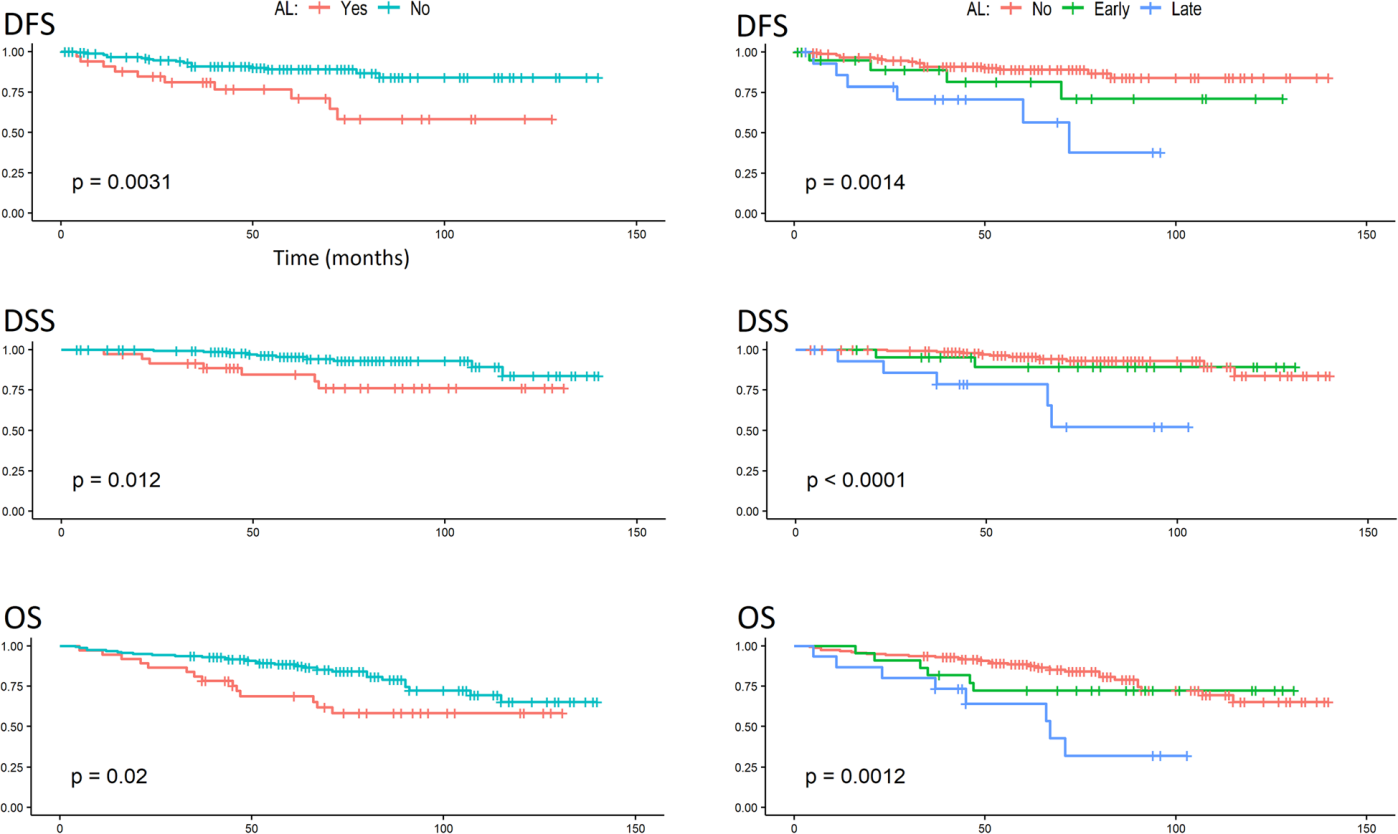
Survival analysis (DFS, DSS, and OS) of patients with ALs in the whole group and with the division into early and late ALs

**Fig. 2:**
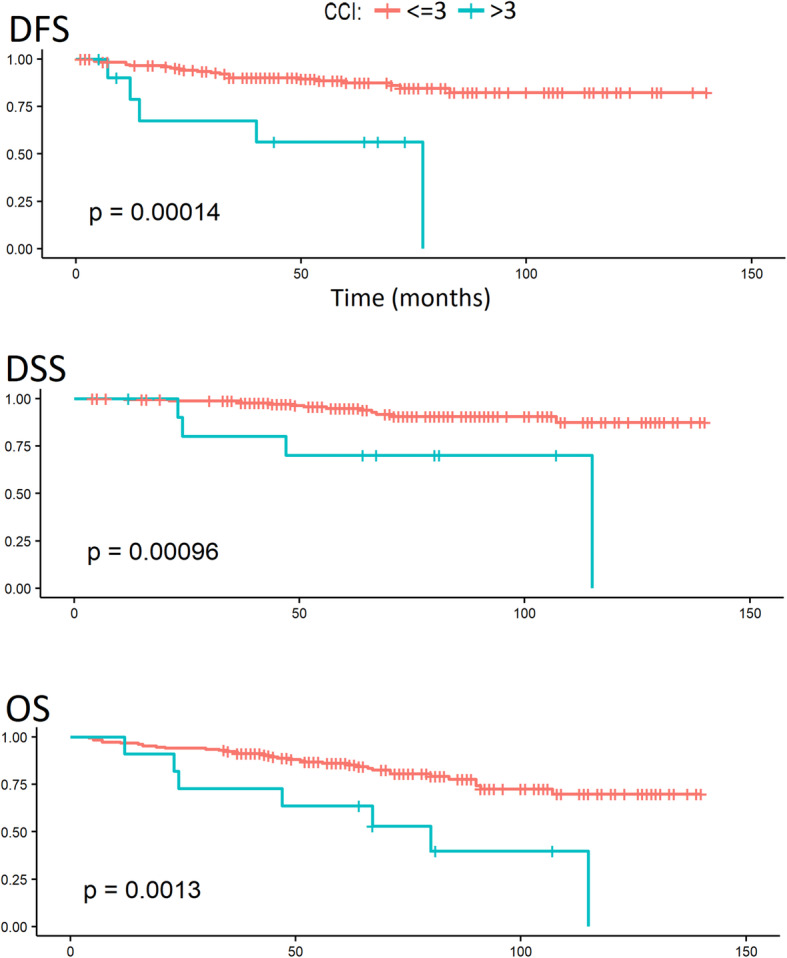
Survival analysis (DFS, DSS, and OS) depending on CCI (≤ 3 vs > 3)

**Fig. 3:**
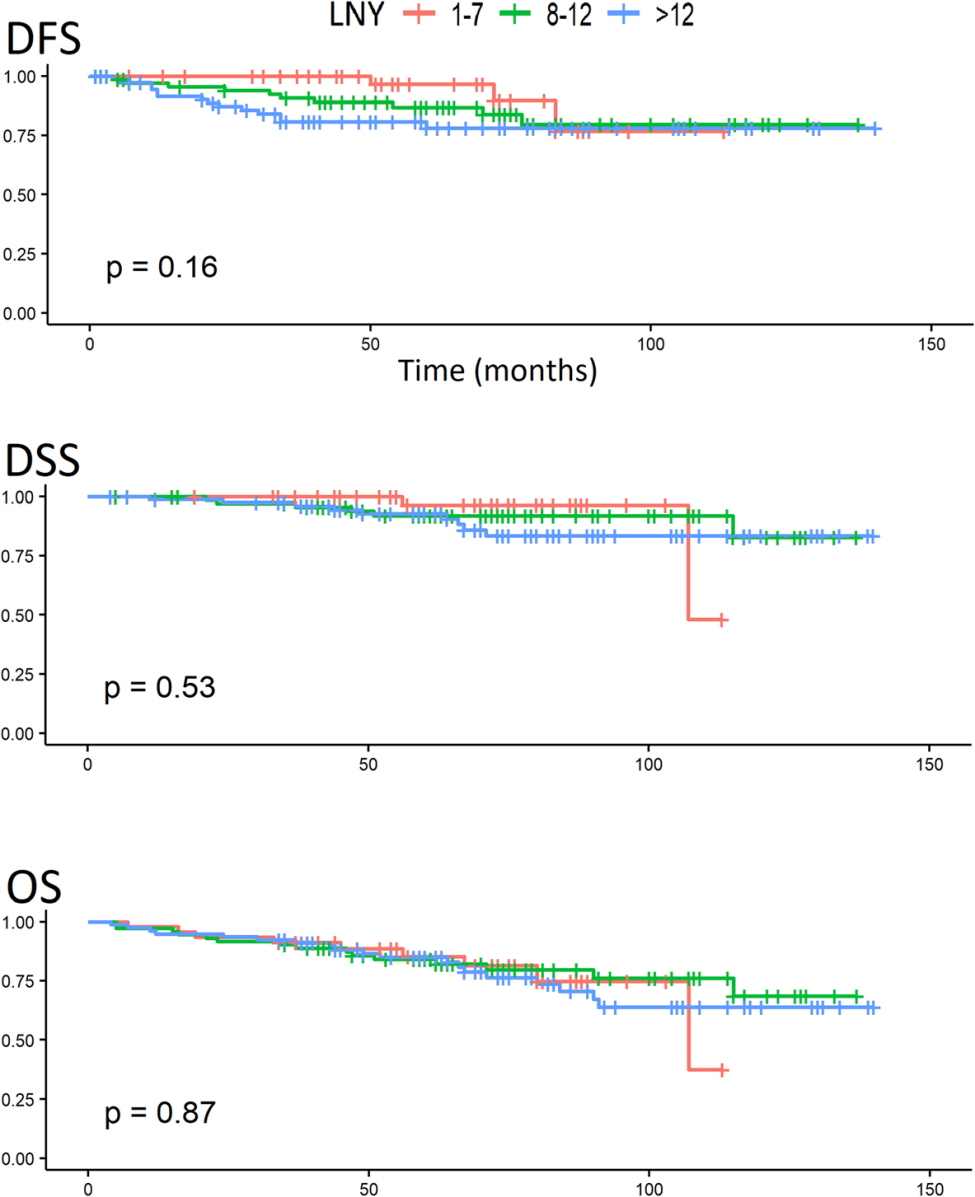
Survival analysis (DFS, DSS, and OS) of patients depending on LNY

In the univariate analysis of survival, the occurrence of AL had a significant impact on DFS, DSS, and OS, as shown by the log-rank test. After the division of ALs into early and late ALs, it was found that only patients with late ALs had a significantly worse prognosis. The probability of survival depending on the occurrence of AL is given in Fig. 1. Patients with CCI ≤ 3 had a significantly better prognosis compared to patients with CCI > 3 in terms of DFS, DSS, and OS. The probability of survival depending on CCI is given in Fig. 2. Table 3 lists the 3- and 5-year survival probabilities depending on the above factors. No relationship was found between survival and other parameters, including LNY. The probability of survival depending on LNY is given in Fig. 3.

**Table 3:** Three- and 5-year survival probabilities depending on the parameters significant in the univariate analysis

	DFS		DSS		OS	
	3 years%	5 years%	3 years%	5 years%	3 years%	5 years%
Total study group	89.0	85.7	97.9	93.4	91.3	84.8
No AL	90.8	88.9	99.3	95.3	93.7	88.4
AL	80.9	71.2	91.5	84.3	81.1	68.6
Early AL	88.8	81.4	95.2	89.3	81.8	72.2
Late AL	70.7	56.6	85.7	77.9	80.0	63.6
CCI ≤ 3	90.2	87.4	98.3	94.7	92.4	86.0
CCI > 3	67.5	56.2	80.0	70.0	72.7	63.6

*DFS* disease-free survival, *DSS* disease-specific survival, *OS* overall survival, *AL* anastomotic leakage, *CCI* Charlson comorbidity index

The results of the univariate and multivariate Cox regression analyses are presented in Table 4. In the multivariate analysis, CCI > 3 was a significant risk factor for DFS (HR 5.78, 95% CI 2.15–15.51, *p* < 0.001), DSS (HR 7.25, 95% CI 2.25–23.39, *p* < 0.001), and OS (HR 3.9, 95% CI 1.72–8.85, *p* = 0.001). Similarly, the occurrence of late AL had a significant negative impact on the risk of DFS (HR 5.05, 95% CI 1.97–12.93, *p* < 0.001), DSS (HR 10.84, 95% CI 3.44–34.18, *p* < 0.001), and OS (HR 4.3, 95% CI 1.94–9.53, *p* < 0.001). No significant influence of early AL or other factors on long-term survival was found.

**Table 4:** Results of univariate and multivariate Cox regression analyses

		Univariate				Multivariate			
		HR	CI 2.5%	CI 97.5%	*p*	HR	CI 2.5%	CI 97.5%	*p*
DFS	CCI ≤ 3	Ref.							
	CCI > 3	5.438	2.043	14.48	**< 0.001**	5.779	2.153	15.51	**< 0.001**
	Female	Ref.							
	Male	2.19	0.9255	5.184	0.0744				
	PRETR ST II	Ref.							
	PRETR ST III	0.6896	0.3156	1.506	0.3512				
	Dist av 1–5 cm	Ref.							
	6–10 cm	0.6141	0.2494	1.512	0.2889				
	11–15 cm	1.269	0.4858	3.313	0.6269				
	ypT0	Ref.							
	ypT1–2	1.888	0.2451	14.55	0.5417				
	ypT3	3.031	0.3978	23.1	0.2845				
	LNY 1–7	Ref.							
	LNY 8–12	2.106	0.5791	7.66	0.2581				
	LNY > 12	3.106	0.8916	10.82	0.0751				
	No AL	Ref.							
	AL	3.066	1.403	6.702	**0.005**				
	No AL	Ref.							
	Early AL	2.001	0.6725	5.953	0.2125	1.96	0.6587	5.831	0.2265
	Late AL	4.753	1.87	12.08	0.0011	5.051	1.974	12.93	**< 0.001**
	No adj CT	Ref.							
	Adj CT	0.695	0.1632	2.959	0.6225				
DSS	CCI ≤ 3	Ref.							
	CCI > 3	5.39	1.754	16.56	**0.0033**	7.252	2.249	23.39	**< 0.001**
	Female	Ref.							
	Male	1.543	0.54	4.41	0.418				
	PRETR ST II	Ref.							
	PRETR ST III	0.643	0.2443	1.692	0.3711				
	Dist av 1–5 cm	Ref.							
	6–10 cm	0.5699	0.1834	1.771	0.3309				
	11–15 cm	1.458	0.4378	4.855	0.5392				
	ypT0	Ref.							
	ypT1–2	0.7613	0.0883	6.565	0.804				
	ypT3	2.153	0.2754	16.83	0.4648				
	LNY 1–7	Ref.							
	LNY 8−12	1.605	0.3207	8.033	0.5648				
	LNY > 12	2.267	0.4854	10.59	0.2979				
	No AL	Ref.							
	AL	3.218	1.222	8.476	**0.018**				
	No AL	Ref.							
	Early AL	1.245	0.2705	5.73	0.7785	1.543	0.3303	7.21	0.5812
	Late AL	8.519	2.825	25.69	**< 0.001**	10.84	3.435	34.18	**< 0.001**
	No adj CT	Ref.							
	Adj CT	0.4898	0.0642	3.735	0.4911				
OS	CCI ≤ 3	Ref.							
	CCI > 3	3.494	1.553	7.859	**0.0025**	3.899	1.718	8.85	**0.0011**
	Female	Ref.							
	Male	0.984	0.5321	1.82	0.9591				
	PRETR ST II	Ref.							
	PRETR ST III	0.6993	0.3791	1.29	0.2522				
	Dist av 1–5 cm	Ref.							
	6–10 cm	0.5456	0.281	1.06	0.0736				
	11–15 cm	0.7257	0.3136	1.679	0.4538				
	ypT0	Ref.							
	ypT1–2	1.258	0.2893	5.466	0.7598				
	ypT3	2.332	0.5488	9.905	0.2514				
	LNY 1–7	Ref.							
	LNY 8–12	0.8853	0.3847	2.037	0.7746				
	LNY > 12	1.064	0.4784	2.365	0.8798				
	No AL	Ref.							
	AL	2.103	1.109	3.987	**0.0227**				
	No AL	Ref.							
	Early AL	1.292	0.5347	3.123	0.5691	1.415	0.5835	3.431	0.4425
	Late AL	3.943	1.792	8.677	**< 0.001**	4.298	1.939	9.526	**< 0.001**
	No adj CT	Ref.							
	Adj CT	0.8261	0.2933	2.327	0.7177				

Bold values indicate statistical significance

*DFS* disease-free survival, *DSS* disease-specific survival, *OS* overall survival, *HR* hazard ratio, *CI* confidence interval, *CCI* Charlson comorbidity index, *Dist av* distance from the anal verge, *PRETR ST* pretreatment clinical stage, *RT* radiotherapy, *LNY* lymph node yield, *AL* anastomotic leakage, *Adj CT* adjuvant chemotherapy

## Discussion

Despite the influence of ALs on long-term oncologic outcomes [4, 5, 17], some reports did not confirm such a relationship [6, 18, 19]. There are several hypotheses in the literature which explain the possible mechanisms of AL-induced cancer relapse [20]. It was shown that during the resection procedure, exfoliated malignant cells which are present in the lumen of the colon have the potential to be implanted into the surrounding tissues. In the case of AL, these cells can penetrate beyond the lumen of the intestine and initiate secondary tumor foci [21]. Another hypothesis highlights the role of acute phase factors and inflammatory mediators in tumor progression and metastasis. In vitro studies found that the peritoneal fluid collected from patients with ALs or from patients with other inflammatory processes in the abdominal cavity resulted in an increase in migration and invasion of cancer cell lines [22]. Additionally, both circulating cancer cells and immune cells show the tendency to migrate to inflammatory sites, thus enhancing the cascade of angiogenesis and proliferation. Impossibility to administer adjuvant chemotherapy or deferral of such therapy is a possible indirect mechanism of the impact of AL on survival. However, this mechanism seems less likely in this case, given the characteristics of the low-risk group and the lack of impact of adjuvant chemotherapy in our analysis. We demonstrated that only late ALs had an adverse effect on survival. Late ALs are an underestimated clinical problem in rectal cancer surgery. More than 50% of ALs may occur after hospital discharge, whereas 25–40% may occur after 30 days following surgery [23, 24]. Definitions of late AL are different, depending on the authors. The common criterion is a period of over 30 days after surgery. However, a period of over 90 days and a less precise determination of AL after hospital discharge were also reported [23]. In accordance with the criterion we adopted, late ALs accounted for 40.5% of all ALs in our material. Late AL is more prevalent in patients with LI, which can be explained by a delay in the diagnosis of AL [25]. It was not confirmed in our material. However, this may be due to the relatively low rate of LI in our patients. The etiopathogenesis of late AL has not yet been elucidated. According to some reports, patient-dependent factors such as the severity of comorbidities or past RT, which may adversely affect the healing process, play a role in late ALs, as opposed to early ALs, where risk factors are mainly those that influence the course of surgery [24, 26] It was found that late ALs were more asymptomatic compared to early ALs. They were more prevalent in the form of fistulas and did not often require radical surgical intervention and became chronic over time. Chronic presacral sinus formation was more commonly found in late ALs (even in 65% of cases) [27].

To the best of our knowledge, there are no reports in the literature that could explain the adverse effect of late ALs with a simultaneous lack of impact of early ALs on survival. Considering that the course of late ALs is usually chronic, it is possible that the duration of influence of the pathogenetic factor can play an important role. However, this is only our hypothesis. More research is needed on the pathogenesis of late ALs in terms of their impact on survival.

We demonstrated a negative effect of CCI > 3 on DFS, DSS, and OS. The influence of comorbidities on the survival of cancer patients may result from several mechanisms. Comorbidities increase the risk of death during the follow-up for reasons other than cancer. They also limit the possibility of optimal treatment (e.g., adjuvant systemic treatment) and may also directly affect tumor progression. While the first two causes are evident and have an established impact on OS, the mechanism of the direct influence of comorbidities is still unclear, although the problem has been raised for a long time [28, 29]. Diabetes mellitus is the only disease in which a direct impact on DFS was confirmed in locally advanced and disseminated colorectal cancer, regardless of systemic treatment. A direct interaction between diabetes and the progression of colorectal cancer is associated with hyperinsulinemia, an increase in insulin-like growth factor, hyperglycemia, and inflammation [30]. Comorbidities may result in the exclusion of patients from adjuvant therapy. In our analysis, most patients did not require standard adjuvant therapy. Only 8.2% of patients underwent such therapy. However, we demonstrated the impact of CCI on DFS without the simultaneous influence of adjuvant chemotherapy on the probability of survival. Therefore, it seems that in ypN0 patients, the influence of CCI on DFS can be the result of the direct influence of comorbidities on the course of cancer disease. However, the mechanism of this interaction remains unknown. Baretti et al. showed that the presence of comorbidities assessed by CCI had a significant negative impact on DFS and OS in stage II/III colorectal cancer patients. However, they did not perform the analysis of individual stages or locations [11]. The influence of CCI on DFS and OS in patients with stages I–III colorectal cancer and different tumor location was demonstrated by Yamano et al. who did not find the influence of CCI on DFS in patients with stage II [31]. The mechanism by which CCI affects DSS can be complex and can be related to a direct influence of comorbidities on tumor progression. Additionally, it should be noted that in patients with active cancer disease, death may also occur due to diseases other than cancer. However, it can be reported as cancer-related death in medical records, which may distort the results of the analyses.

It has been shown that preoperative nodal understaging in rectal cancer patients may be related to approximately 15% of patients compared to histological assessment. However, neither the effect of the above finding nor the impact of clinical nodal staging on long-term outcomes was shown [32, 33]. We did not find such an impact, although our analysis was conducted on a heterogeneous group of patients (cT3N0, cT1-3N+). Additionally, we did not demonstrate the prognostic influence of LNY. Studies confirmed the effects of ionizing radiation on lymph nodes, including stromal atrophy and fibrosis, as well as lymphocyte count reduction [34, 35]. Preoperative radiotherapy was shown to reduce the total number of removed lymph nodes [7, 12]. Of note, studies found the reduction in the number of lymph nodes due to the response to neoadjuvant therapy associated with the response of the immune system [36]. Studies on colon cancer patients found that despite the tendency to increase the number of removed nodes, the percentage of patients with metastatic lymph nodes did not increase [37, 38]. It was also shown that fat clearance increased the median number of retrieved lymph nodes in ypN0 rectal cancer patients from 12 to 19.5 compared to the conventional fixation method. However, it did not affect the long-term outcomes [39]. Thus, technical factors (both surgical and histological) seem to be of secondary importance in terms of understaging. Despite the above, attempts are made to establish the minimum number of lymph nodes below which a risk of understaging is observed and to determine a group of patients who might benefit from adjuvant treatment due to low LNY. The results of the studies are contradictory. It should be noted that most analyses reporting the cut-off point below which the understaging is found in ypN0 patients were based on single-center studies with a low (≤ 7) median number of removed lymph nodes in the entire group [40, 41] or were based on the data from the national multicenter registries [8, 9, 42, 43]. Nevertheless, many studies indicated that LNY had no effect on understaging and thus on long-term survival in ypN0 patients, which is in line with our findings [10, 11, 44–46]. The results of our analysis may confirm the theory that LNY in ypN0 patients should be considered in terms of the response to neoadjuvant therapy rather than as a determinant of the quality of surgical or histological procedures with no impact on long-term outcomes.

The analysis has limitations typical of retrospective and single-center analyses. CCI was assessed retrospectively based on medical record data. In addition, the strength of inference may be reduced by the fact that the study group is a predefined low-risk group.

## Conclusions

Late AL and CCI > 3 are the factors that may influence long-term oncologic outcomes in ypN0M0 rectal cancer patients after neoadjuvant therapy and AR. No evidence of the impact of LNY on understaging was found.

## Supplementary Information

**Additional file 1.** Flowchart showing the formation of the study group

**Additional file 2.** Charlson comorbidity index

## Data Availability

All data generated or analyzed during this study are included in this published article [and its supplementary information files].

## Abbreviations

AL: Anastomotic leakage
AR: Anterior rectal resection
CCI: Charlson comorbidity index
CRT: Chemoradiotherapy
DFS: Disease-free survival
DSS: Disease-specific survival
G: Tumor grade
HR: Hazard ratio
ISREC: International Study Group of Rectal Cancer
LI: Loop ileostomy
LNY: Lymph node yield
LVI: Lymphovascular invasion
OS: Overall survival
PNI: Perineural invasion
RT: Radiotherapy
SD: Standard deviation
TRG: Tumor regression grade
