# Supplementary material for: The assessment of risk factors for long-term survival outcome in ypN0 patients with rectal cancer after neoadjuvant therapy and radical anterior resection
Source: World J Surg Oncol. 2021 May 21;19:154. doi: 10.1186/s12957-021-02262-x (PMC8140444; doi:10.1186/s12957-021-02262-x)
Supplement: Supplementary file 1 — Additional file 1. Flowchart showing the formation of the study group [file 12957_2021_2262_MOESM1_ESM.docx]

(-)

(-)

(-)

(-)

Radical anterior resections after RT or CRT without major risk factors - STUDY GROUP

n= 195

ypN+ n= 119

Mucous component n=3

PNI and/or LVI n=6

Postoperative deaths n=5

Radical anterior resections after RT or CRT

n= 328

No neoadjuvant treatment

n=128

Radical anterior rectal resections

n= 456

APR n=267

Hartmann’s procedure n=52

Local excision n=28

Radical resections

n= 803

Patients with dissemination (M1) n=103

Non-radical (R1/R2) resections, including CRM+ n=15

Patients who underwent surgery for rectal cancer (2008-2016)

n= 921

CRM- circumferential radial margin, APR- abdominoperineal resection, RT- radiotherapy, CRT- chemoradiotherapy, PNI- perineural invasion, LVI- lymphovascular invasion
