# Supplementary material for: The assessment of risk factors for long-term survival outcome in ypN0 patients with rectal cancer after neoadjuvant therapy and radical anterior resection
Source: World J Surg Oncol. 2021 May 21;19:154. doi: 10.1186/s12957-021-02262-x (PMC8140444; doi:10.1186/s12957-021-02262-x)
Supplement: Supplementary file 2 — Additional file 2. Charlson comorbidity index [file 12957_2021_2262_MOESM2_ESM.docx]

Charlson comorbidity index scoring:

myocardial infarct (1), congestive heart failure (1), peripheral vascular disease (1), cerebrovascular disease (1), dementia (1), chronic pulmonary disease (1), connective tissue disease (1), peptic ulcer disease (1), mild liver disease (1), diabetes (1), hemiplegia (2), moderate or severe renal disease (2), diabetes with end organ damage (2), any tumor (2), leukemia (2), lymphoma (2), moderate or severe liver disease (3), metastatic solid tumor (6), AIDS (6).

Based on:

Charlson ME, Pompei P, Ales KL, MacKenzie CR. A new method of classifying prognostic comorbidity in longitudinal studies: development and validation. J Chron Dis. 1987;40(5):373–83. DOI: 10.1016/0021-9681(87)90171-8.
